# Laparoscopic versus Open Transverse-Incision Approach for Right Hemicolectomy: A Systematic Review and Meta-Analysis

**DOI:** 10.3390/medicina57010080

**Published:** 2021-01-19

**Authors:** Claudio F. Feo, Panagiotis Paliogiannis, Alessandro Fancellu, Angelo Zinellu, Giorgio C. Ginesu, Carlo V. Feo, Alberto Porcu

**Affiliations:** 1 Unit of General Surgery 2, Department of Medical, Surgical and Experimental Sciences, University of Sassari, 07100 Sassari, Italy; afancel@uniss.it (A.F.); ginesugc@uniss.it (G.C.G.); alberto@uniss.it (A.P.); 2 Department of Biomedical Sciences, University of Sassari, 07100 Sassari, Italy; panospaliogiannis@gmail.com (P.P.); azinellu@uniss.it (A.Z.); 3 Unit of General Surgery, Azienda USL di Ferrara, Department of Medical Sciences, University of Ferrara, 44121 Ferrara, Italy; cvfeo@unife.it

**Keywords:** colon cancer, right colectomy, laparoscopy, laparotomy, transverse incision, open surgery

## Abstract

*Background and Objectives:* There is general agreement on the benefits of laparoscopy for treatment of rectal and left colon cancers, whereas findings regarding the comparison of laparoscopic and open right colonic resections are discordant. The aim of this systematic review and meta-analysis was to assess the outcomes and advantages of laparoscopic versus transverse-incision open surgery for management of right colon cancer. *Materials and Methods:* A systematic review was performed according to the Preferred Reporting Items for Systematic Review and Meta-Analyses (PRISMA) guidelines. Comparative studies evaluating the results of laparoscopic and transverse-incision open right hemicolectomies were analyzed. The measured outcomes were mean operative time, time to feeding, duration of hospital stay, and number of lymph nodes harvested. *Results:* A total of 5 studies including 318 patients met the inclusion criteria. Meta-analysis revealed no differences in time to resume oral feeding, hospital stay, and number of lymph nodes harvested in between groups, but mean length of surgery was significantly longer in the laparoscopic group. *Conclusion:* These data confirm that the preferred approach to right hemicolectomy is yet unclear. Laparoscopy has a longer operative time than transverse-incision open surgery, and no significant short-term benefits were observed for the studied parameters. Well-designed randomized control trials (RCTs) might help to identify the differences between these two techniques for the surgical treatment of right colon cancer.

## 1. Introduction

The advantages of laparoscopy for rectal and left-sided cancers are well documented [1,2,3,4], but the optimal surgical technique for right colectomy is still unclear [5,6]. While laparoscopic rectal resection and left colectomy are increasing over time, laparoscopic right hemicolectomy has a very slow diffusion all over the world. In a recent analysis of 4997 patients operated on from 2010 to 2019 in Germany for right-sided colon cancer, the procedures were performed laparoscopically only in 18.7% of the cases [6]. Only a few studies, nonrandomized and including small populations, have compared laparoscopic to open right colonic resections, with no clear advantage of one approach over the other. Several studies have suggested that a transverse incision rather than a midline laparotomy may enhance the postoperative recovery following open abdominal surgery [7,8,9]. In particular, a transverse laparotomy may offer a few advantages such as less postoperative pain, reduced impact on respiratory function, better cosmetic effect, and lower rate of incisional hernia.

The aim of this systematic review and meta-analysis was to assess the outcomes and advantages of laparoscopic versus transverse-incision right hemicolectomy for the surgical treatment of right colon cancer.

## 2. Materials and Methods

### 2.1. Search Strategy, Eligibility Criteria, and Study Selection

This meta-analysis was performed following the Preferred Reporting Items for Systematic Review and Meta-Analyses (PRISMA) guideline. A systematic electronic search of published items from January 1990 to December 2020 was conducted in the PubMed, Web of Science (WOS), and Scopus repositories, using the following keywords: “right colectomy” AND “laparoscopy” or “right colectomy” AND “laparotomy”. Cross-check of the references of the articles found has also been performed in order to detect further missing papers.

Abstracts were screened independently by two researchers (AF and GCG) to establish relevance. If relevant, the two researchers independently reviewed the full articles. Any disagreement between the reviewers was resolved by a third researcher (CFF). Eligibility criteria were: (i) comparison in patients with right colon cancer treated with laparoscopy or transverse-incision open surgery, (ii) outcomes including mean operative duration, time to oral feeding, hospital length of stay, and/or number of lymph nodes harvested, (iii) adult population, (iv) English language, and (vi) full-text publication.

The Newcastle–Ottawa Scale (NOS) was employed to assess the individual quality of the enrolled studies [10]. This scale evaluates the subsequent issues: (1) cohort selection, (2) cohort comparability, (3) exposure ascertainment modalities, and (4) outcome of interest assessment. NOS scores of 1–3, 4–6, and 7–9 respectively designate poor, intermediate, and high study quality.

### 2.2. Statistical Analysis

Standardized mean differences (SMD) were used to create forest plots of continuous data and to evaluate differences between patient groups. *p* < 0.05 was set as cut-off for statistical significance, and 95% confidence intervals (CIs) were stated. In three studies [11,12,13], the mean and standard deviation values were calculated from median and range or IQR as previously described [14]. Q statistic (significance at *p* < 0.10) was employed to evaluate heterogeneity between studies. In addition, the I^2^ statistic was calculated (I^2^ < 25%, no heterogeneity; I^2^ between 25% and 50%, moderate heterogeneity; I^2^ between 50% and 75%, large heterogeneity; and I^2^ > 75%, extreme heterogeneity) [15,16]. Random or fixed effects model was employed to determine the pooled SMD and corresponding 95% CIs at the occurrence.

Sensitivity analysis was performed sequentially excluding one study at a time in order to evaluate the impact of each individual study on the overall risk estimate [17]. Statistical analyses were carried out with MedCalc for Windows, version 15.4 64 bit (MedCalc Software, Ostend, Belgium) and Stata 14 (STATA Corp., College Station, TX, USA) software.

## 3. Results

### 3.1. Literature Search and Study Selection

**Figure 1:**
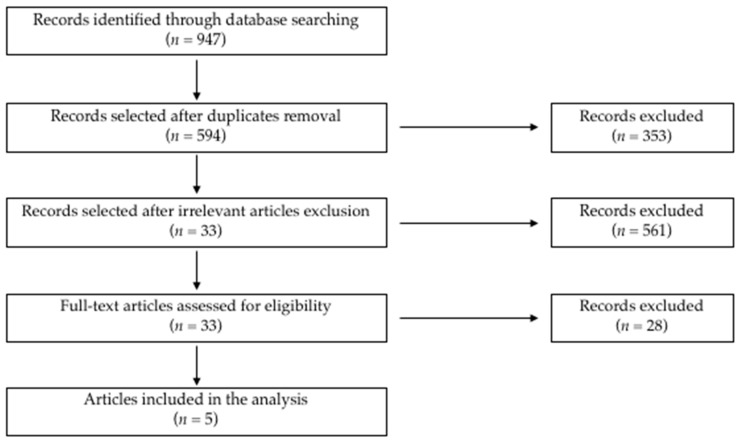
Flowchart illustrating the electronic search strategy and results.

A flowchart depicting the electronic search of the articles is presented in Figure 1. We firstly retrieved 947 potentially relevant studies (PubMed *n* = 375, Scopus *n* = 450, and WOS *n* = 122). A total of 914 studies were subsequently excluded because they were either duplicates (*n* = 353) or irrelevant (*n* = 561). After a full-text evaluation of the remaining articles, 28 further papers were excluded as they did not meet the inclusion criteria. Finally, five studies comprising a total of 318 patients were included in the meta-analysis [11,12,13,18,19]. The characteristics of these studies, published between 2007 and 2019, are summarized in Table 1.

### 3.2. Meta-Analysis of Studied Parameters

**Figure 2:**
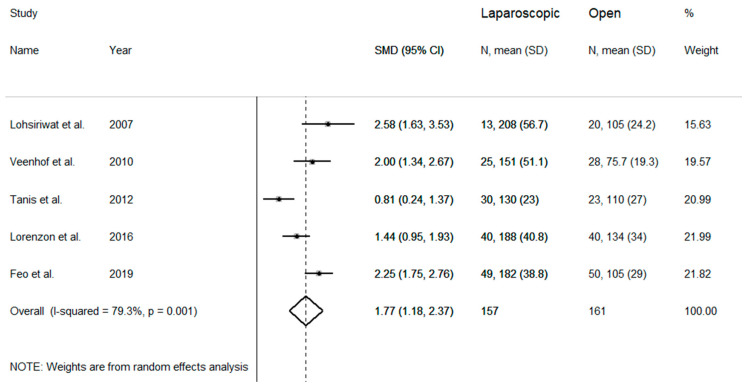
Forest plot for mean operative time.

Mean operative time was evaluated in five studies with substantial heterogeneity (I^2^ = 79.3%, *p* = 0.001), requiring a random-effects model (Figure 2). Pooled results showed that mean operative time was significantly higher in laparoscopic than in open group (SMD = 1.77 min, 95% CI: 1.18 to 2.37 min; z = 5.84 *p* < 0.001). Sensitivity analysis was performed to evaluate results stability. The corresponding pooled SMD values did not significantly change after sequential removal of single studies, with effect size ranging between 1.62 and 2.00 days.

**Figure 3:**
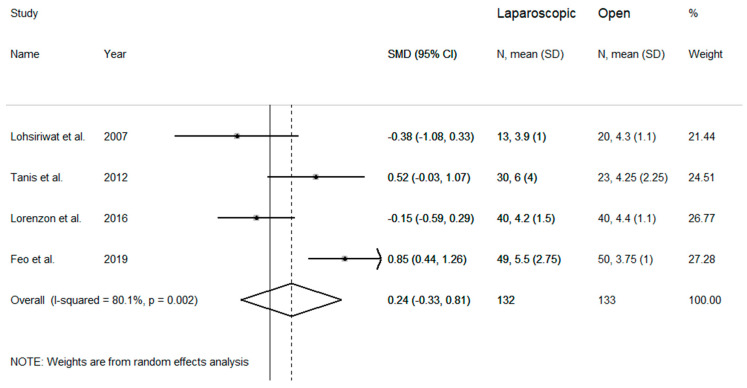
Forest plot for time to feeding.

Time to oral feeding was assessed in four studies showing high heterogeneity (I^2^ = 80.1%, *p* = 0.002), necessitating the use of a random-effects model (Figure 3). No differences were observed between the two studied groups for time to oral feeding (SMD = 0.24 days, 95% CI −0.33 to 0.81 days; z = 0.82 *p* = 0.415). Also in this case pooled SMD were not modified after sequential removal of single studies, with effect size ranging between 0.01 and 0.40 days.

**Figure 4:**
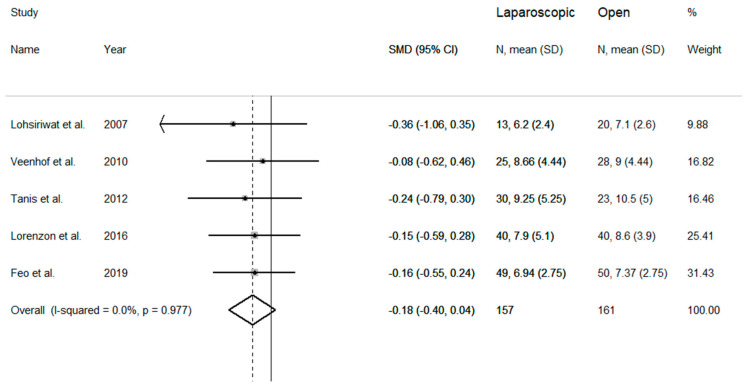
Forest plot for length of hospital stay.

The length of hospital stay was evaluated in five studies (Figure 4). Pooled results showed nonsignificant trends towards shorter duration of hospital stay in the laparoscopic group when compared to the open group (SMD = −0.18 days, 95% CI −0.40 to 0.04 days; z = 1.56 *p* = 0.118). No heterogeneity between studies was observed (I^2^ = 0.00%, *p* = 0.98), therefore the fixed-effects model was used. Pooled SMD values were similar after sequential removal of single studies, with effect size ranging between −0.19 and −0.16 days.

**Figure 5:**
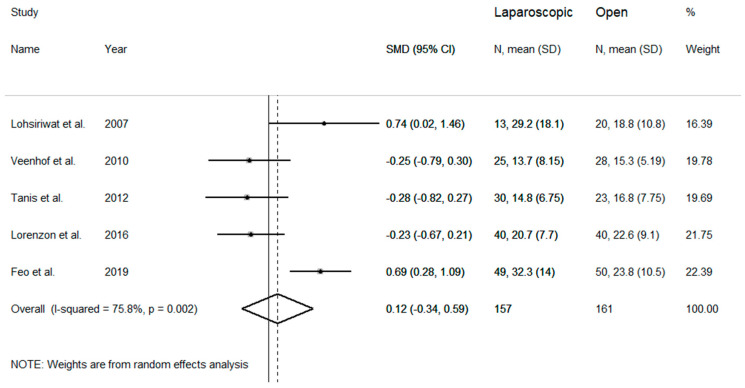
Forest plot for number of lymph nodes harvested.

Number of lymph nodes was assessed in five studies showing elevated heterogeneity (I^2^ = 75.8%, *p* = 0.002), which required the use of a random-effects model (Figure 5). No difference was observed between the number of lymph nodes of the two studied groups (SMD = 0.12, 95% CI −0.34 to 0.59; z = 0.51 *p* = 0.607). Pooled SMD did not vary after sequential removal of single studies, with effect size ranging between −0.06 and 0.22.

Publication bias was not assessed because of the limited number of studies.

## 4. Discussion

In recent years, there has been an increasing widespread use of laparoscopic techniques for the management of patients with colorectal cancer. In particular, considering laparoscopic resections of the left colon and rectum, the postoperative recovery is accelerated with comparable oncological outcomes to those of the open approach [1,2,3,4]. These issues, however, are less clear when laparoscopic and open techniques are applied to the right colon resections.

Santoro et al. [20] performed a systematic review in 2014 analyzing 350 patients who underwent a right hemicolectomy, comparing transverse-incision laparotomy (*n* = 141), vertical midline laparotomy (*n* = 104), and laparoscopic approach (*n* = 105). The authors concluded that the studies analyzed ranged from small randomized control trials (RCTs) to retrospective series creating a heterogeneous sample, with no real significant differences between the three techniques. Arezzo et al. [21] published a meta-analysis in 2015 evaluating differences in safety of laparoscopic and open right colectomy. They analyzed 26 studies for a total of 3307 patients: there were 24 non-RCTs (3096 patients) and only 2 RCTs (211 patients), considering both benign and malignant diseases. The authors found that mortality and morbidity (primary outcomes) were significantly lower after laparoscopy than open surgery. Moreover, most of the secondary endpoints such as use of narcotics, day of first flatus, resumption of oral intake, blood loss, wound infection, and duration of hospital stay favored laparoscopy. However, operating time was significantly shorter in the open group. Very recently, Jurowich et al. [6] analyzed 4997 patients undergoing oncological right hemicolectomy, retrieved from the German StuDoQ|ColonCancer registry; 4062 (81.3%) underwent an open and 935 (18.7%) a laparoscopic procedure. Patients operated on laparoscopically were significantly younger and had lower American Society of Anesthesiologist (ASA) scores, as well as fewer and less severe comorbidities. Patients in the laparoscopic group had a significantly reduced hospital stay, whereas those in the open group had a significantly shorter operating time and a higher number of lymph nodes harvested. Therefore, the authors concluded that no relevant advantages could be found for the minimally invasive approach.

Unfortunately, however, in the reports from Arezzo [21] and Jurowich [6], it is not specified what type of incision was performed in open right hemicolectomies and, presumably, most patients received a midline laparotomy. Arezzo found several short-term advantages for laparoscopic right colectomy, although the majority of data were retrieved from non-RCTs and probably patients with greater illness severity were treated with an open access rather than laparoscopy. Surprisingly, these advantages in favor of laparoscopy were not confirmed in the analyses from Jurowich, who analyzed a greater number of patients of whom those in the laparoscopic group were younger and with a better performance status.

Rausa et al. [22] have published in 2019 a network meta-analysis comparing open, laparoscopic-assisted, total laparoscopic, and robotic right hemicolectomy for either malignant or benign disease. Data from 5 RCTs and 25 retrospective and 18 prospective controlled studies were analyzed for a total of 5652 patients. The authors found that risk of postoperative complications and surgical site infection rate after total laparoscopic and robotic procedures were significantly lower compared to laparoscopic-assisted and open approaches. However, reoperations rate, 30-day mortality, 60-day readmission rate, and number of lymph node retrieved were comparable across the four surgical modalities. They concluded that both robotic and total laparoscopic operations have considerable benefits regarding perioperative outcomes compared to the other two surgical techniques. Again, in this meta-analysis, most data come from non-RCTs and, as stated in the discussion, the reason why each patient received a specific surgical approach was not reported, which may represent a selection bias. Moreover, in all but one study, open right hemicolectomy was performed via a midline laparotomy which may well have influenced postoperative short-term outcomes.

A retrospective comparative study [23] of patient-reported outcomes in laparoscopic and open right hemicolectomy for colon cancer was performed in Canada in 2019. A total of 1022 patients were analyzed and no difference in the percentage of patients with moderate-to-severe symptom scores was observed between laparoscopic and open procedures. A meta-analysis published in 2020 [24] analyzed 26 studies (*n* = 3410) which compared intra- and postoperative complications after laparoscopic (*n* = 1515) and open (*n* = 1895) surgery for right-sided colon cancer. Most data were from observational studies and the authors concluded that postoperative outcomes were almost comparable between the two surgical techniques. Finally, another very recent meta-analysis [25] compared hand-assisted laparoscopic (*n* = 238) and open (*n* = 268) right hemicolectomy for colon cancer. Five of the seven studies included in the analysis were non-RCTs for a total of 506 patients. The authors found that hand-assisted laparoscopic surgery was superior to the open approach in terms of postoperative recovery with similar oncological outcomes. Of note, none of the open surgery procedures was performed via a transverse-incision laparotomy, which was the approach elected in our investigation.

It has already been demonstrated that transverse-incision colectomy may reduce postoperative pain, improve postoperative recovery, and shorten length of hospital stay [26,27]. Therefore, we elected to restrict our systematic review with meta-analysis to comparative studies evaluating exclusively the results of laparoscopic as opposed to transverse-incision open right hemicolectomies. We found five retrospective comparative studies on this topic, including a total of 318 patients submitted to right hemicolectomy for colon cancer in the majority of cases; 157 were treated with laparoscopy and 161 received a transverse laparotomy. There were no differences in between groups regarding the time to resume oral feeding, length of stay, and number of lymph nodes retrieved, but the mean operative time was significantly longer in the laparoscopic group. Most patients in the laparoscopic group (four out of five studies) received a mini-laparotomy for specimen removal and extracorporeal suturing. Presumably, the short incision of laparoscopic patients operated on with an extracorporeal anastomosis technique was only slightly shorter than the transverse laparotomy performed in open patients, which might help to explain why no short-term benefits were found in between groups. It could be argued that total laparoscopic and robotic right hemicolectomy with a small suprapubic incision may offer postoperative advantages over the other more invasive techniques, including the transverse-incision open approach. This issue, however, has not yet been clarified in the current literature.

Our study has some limitations to be considered. First, there was a limited number of patients retrieved from a small number of retrospective studies and no RCTs; therefore, publication bias could not be assessed. Second, in one study, no details were available about the perioperative care protocol adopted for patients’ recovery, which may well influence the postoperative outcomes that were analyzed. Third, in the laparoscopic group patients, the learning curve period to master the technique might have been included as opposed to open surgery. Furthermore, in most comparisons, a consistent heterogeneity among the studies has been observed. On the other hand, the present meta-analysis is the first to evaluate the outcomes of the open transverse-incision and the laparoscopic approaches for right colon cancer resections, on the basis of the current literature.

## 5. Conclusions

Our data confirm that the preferred approach to right hemicolectomy is yet unclear. Laparoscopy has a longer operative time than transverse-incision open surgery, and no significant short-term benefits were observed for the studied parameters. Well-designed RCTs might help to identify the differences between these two techniques for the surgical treatment of right colon cancer.

## Figures and Tables

**Table 1:** Characteristics of the studies included in the systematic review and meta-analysis.

Authors and Publication Year	Country and Study Period	Type	No.	Lap	Open	Gender (M/F)		Age (Mean ± SD or Median and Range)		Anastomosis
						Lap	Open	Lap	Open	
Lohsiriwat et al., 2007	Thailand 2004–2006	full-text	33	13	20	6/7	7/13	56.9 ± 13.5	65.2 ± 16.0	EA
Veenhof et al., 2011	The Netherlands 2005–2009	full-text	53	25	28	13/12	9/19	68 (61–69)	75 (67–78)	N/A
Tanis et al., 2012	The Netherlands 2006–2009	full-text	53	30	23	12/18	10/13	75 (31–85)	73 (54–85)	EA
Lorenzon et al., 2016	Italy 2005–2014	full-text	80	40	40	17/23	20/20	70.4 ± 9.2	71.4 ± 11.9	EA
Feo et al., 2019	Italy 2013–2016	full-text	99	49	50	23/26	24/26	69.0 (40–84)	70.0 (50–82)	EA

LAP laparoscopy, OPEN laparotomy, EA extracorporeal anastomosis, N/A not available.
